# The ‘Designer’ Artificial Pancreas: How Does Current Technology Mimic Pancreatic Physiology?

**DOI:** 10.7759/cureus.86770

**Published:** 2025-06-25

**Authors:** Lucie Ayliffe Daly

**Affiliations:** 1 Emergency, Royal Brisbane and Women's Hospital, Herston, AUS

**Keywords:** artificial pancreas, closed-loop feedback, diabetes, diabetes management, hybrid, insulin pump, type 1 diabetes

## Abstract

Type 1 diabetes (T1D) is characterised by the autoimmune destruction of pancreatic insulin-producing beta cells, leading to a lifelong dependency on exogenous insulin administration for survival. Tight glycaemic control is essential to reduce the risk of long-term complications but achieving this is challenging due to high variability in insulin requirements, creating a significant burden for individuals and families.

Automated insulin delivery systems, which detect real-time blood glucose levels and self-adjust insulin delivery rates, have long been a goal in the management of diabetes. These systems aim to mimic healthy endocrine pancreatic physiology by replicating the glucose-responsive feedback control, thereby improving glycaemic outcomes and reducing the burden of self-management.

Over the last decade, several technological advances have culminated in the development of the artificial pancreas system (APS). Since the release of the first commercially available ‘hybrid’ system in 2017, multiple innovations have improved APS accuracy, personalisation and user experience.

This review discusses the evolution of the APS and evaluates how hybrid, closed-loop and dual-hormone systems replicate pancreatic physiology. We assess clinical and psychosocial outcomes and consider what future technological advancements, such as nuanced insulin analogues, alternative delivery routes, and machine learning algorithms, might enable a truly “designed” APS.

## Introduction and background

Type 1 diabetes (T1D) is characterised by the autoimmune destruction of pancreatic insulin-producing beta (β) cells and lifelong dependency on exogenous insulin for survival [1]. Insulin is an anabolic hormone which acts to maintain normal blood glucose levels by promoting cellular glucose uptake and glycogen synthesis. Insufficient insulin results in hyperglycaemia, and individuals with T1D require multiple daily insulin injections or continuous subcutaneous insulin infusion (CSII) via an insulin pump to maintain normoglycemia [1]. 

The importance of tight glycaemic control was underscored by the landmark 1993 ‘Diabetes Control and Complications Trial’, which demonstrated that intensive insulin therapy significantly reduced the risk of long-term microvascular complications [2]. However, achieving tight control remains challenging due to high variability in insulin requirements [3]. As such, doses must be carefully adapted based on diet, physical activity and lifestyle, which in turn places a considerable burden on individuals and families [4]. This challenge is compounded by the increased risk of hypoglycaemia associated with intensive insulin therapy [5], meaning that even individuals who actively manage their diabetes often fail to achieve optimum glycemic targets.

The ideal therapeutic approach would be one that preserves glycaemic stability whilst minimising manual input, thereby replicating the hormone-regulated feedback loops of healthy pancreatic endocrine function. Such systems have been under development since the 1970s [6], but only within the past decade, with rapid improvements in continuous glucose monitoring technology, have closed-loop systems (CLS) and APSs become clinically viable options.

Early devices operated to suspend insulin delivery at predefined thresholds, but newer systems enable modulation of basal insulin dosing in response to real-time glucose trends and even incorporate glucagon to prevent hypoglycaemia. In this review, we examine the current landscape of APS technologies, how closely they mimic pancreatic endocrine physiology, and what is still needed to build the optimal ‘designer’ APS in the future. 

Components of the artificial pancreas 

The core technology of the artificial pancreas (AP) comprises three components: a continuous glucose monitor (CGM), an insulin pump, and a control algorithm that serves as the system’s decision-making algorithm. These are integrated to combine real-time glucose measurements with continuous subcutaneous insulin infusion (CSII), aiming to maintain glucose levels at a prespecified target and mimic β-cell function [7]. 

A CGM system consists of a subcutaneous needle-type electrode which transmits signals to a receiver, providing a real-time display of interstitial glucose levels [8,9]. Since the FDA approval of the first commercial CGM system in 1999, accuracy has improved substantially [10,11]. Current generation sensors, such as Dexcom G7 and Abbott’s FreeStyle Libre 3 (both approved in 2022-2023), now offer error rates (Mean Absolute Relative Difference - MARD) under 9% and require no calibration [12,13]. Additionally, they enable 10-14 day wear and feature low-profile designs, making them both more accurate and user-friendly than their predecessors. 

Insulin pumps deliver fast-acting insulin analogues via continuous subcutaneous infusion. Doses consist of a basal delivery, to maintain the 24-hour glucose profile, as well as meal-time boluses to correct for hyperglycaemia [14]. Modern pumps allow basal rates to be programmed to match diurnal variations, and bolus profiles can also be customised with features such as extended or dual-wave delivery [15]. Many modern systems combine pumps with CGM to create “sensor-augmented” pumps, which feature automated insulin suspension in response to pre-specified thresholds of hypoglycaemia [7,16]. 

What distinguishes an APS from earlier automated systems is the incorporation of an adaptive control algorithm which effectively acts as the ‘brain’ of the system [17]. This algorithm continuously adjusts insulin infusion rates in response to real-time glucose concentration [7]. CGM values are transmitted to the algorithm, which calculates appropriate insulin doses to maintain target glucose whilst accounting for variability in individual physiology and lifestyle factors. 

There are two primary types of control algorithms: Proportional Integral Derivative (PID) and Model Predictive Controller (MPC). The PID controller alters insulin delivery according to three variables: current glucose level (proportional component), the area under the curve between the measured and target glucose set points (integral), and the rate of change of measured glucose (derivative) [18]. Meanwhile, the MPC uses a mathematical model to predict future glucose trajectories based on current trends and optimise insulin dosing accordingly [19]. However, the nonlinearity and complexity of blood glucose levels make prediction challenging, and accordingly, newer approaches such as fuzzy logic have been explored [20]. These systems utilise codified clinical decision-making processes to allow more personalised and adaptable insulin dosing, accounting for individual differences in lifestyle, insulin sensitivity and response [21].

Each of these algorithms offers strengths: PID is simple and responsive, MPC offers forecasting of future glucose trajectories, and fuzzy logic replicates human-like decision-making in real-world scenarios. Currently, hybrid systems predominantly utilise MPC-based logic; however, experimental systems increasingly explore nuanced algorithms (e.g., deep reinforcement learning [22]) to further optimise and personalise control.

Pancreatic Physiology

To design an artificial pancreas which effectively mimics its biological counterpart, it is important to understand the physiology of the endocrine pancreas and intra-islet hormonal signalling pathways. In healthy individuals, insulin is secreted by β-cells in response to hyperglycaemia, promoting glucose uptake and storage. By contrast, glucagon is secreted from alpha (α) cells in response to hypoglycaemia and acts to stimulate hepatic glycogenolysis and suppress insulin secretion. In healthy individuals, these opposing intricate feedback loops act to maintain glucose levels within a narrow physiological range. 

In Type 1 diabetes, the selective autoimmune destruction of β-cell results in insulin deficiency. Compounding this, α-cell dysfunction leads to inappropriate elevation of glucagon secretion during hyperglycaemia, and over time, individuals also develop a blunted glucagon response to hyperglycaemia. This dual dysregulation leads to both chronic hyperglycaemia and an increased risk of hypoglycaemic episodes [23].

Therefore, to successfully mimic pancreatic physiology, an artificial pancreas system must accurately regulate basal insulin delivery in response to hepatic glucose production while dynamically responding to fluctuations in glycaemia, particularly after meals. It should minimise the risk of hypoglycaemia, ideally by replicating the counter-regulatory effect of glucagon, and adapt to diurnal variations in insulin sensitivity influenced by factors such as physical activity, sleep, and hormonal changes. 

Current systems address these requirements to varying extents. Hybrid systems modulate basal insulin delivery in real time; however, they rely on user-initiated mealtime boluses. Fully closed-loop systems attempt to automate both basal and bolus insulin delivery without user input. Dual-hormone systems, which remain under investigation in clinical trials, integrate automated insulin delivery with glucagon administration in order to more accurately replicate physiological counter-regulatory mechanisms.

The degree to which these systems can replicate each aspect of pancreatic endocrine physiology forms a key criterion for evaluating clinical performance. However, going beyond glycaemic outcomes, the key consideration is how effectively these systems can reduce disease burden and accommodate lifestyle variation and unpredictability, challenges which the biological pancreas navigates effortlessly.

## Review

Artificial pancreas systems 

Hybrid Systems

Hybrid closed-loop systems (HCLs) represent the most widely used artificial pancreas system in current clinical practice. These systems make micro-modulations to background insulin delivery rates according to real-time glucose levels; however, users are still required to manually deliver insulin boluses to account for additional factors such as exercise and meals. As such, whilst this technology does not achieve full automation, it aims to improve glycaemic stability by auto-regulating basal insulin dosing and thus reduce user burden.

A growing body of evidence supports the efficacy and safety of HCLs across a diverse range of populations. An early randomised crossover study by Murphy et al. (2011) involving 12 pregnant women reported that the use of HCL reduced the risk of hypoglycaemia during 24-hour monitoring compared with standard CSII (0.00 vs 0.3%), whilst maintaining the same percentage of time in the target range (81%). However, despite highlighting greater safety, no significant improvement in mean glucose levels was reported, suggesting a limited impact on hyperglycaemia [24]. 

By contrast, Bergenstal et al. (2016) conducted a larger three-month outpatient study in 124 adults and observed more robust improvements in glycaemic control with HCLs compared to standard insulin therapy. They reported a marked reduction in time spent in both hyperglycaemia (27.4% vs. 24.5%) and hypoglycaemia (5.9% vs. 3.3%), as well as an increase in percentage time in the target range (72.2% vs. 66.7%). Likewise, more recent large-scale clinical trials have demonstrated similar improvements across a range of populations, including pregnant women [25], children [26] and adults with sub-optimally controlled diabetes [27]. A landmark 12-week multicentre trial by Tauschmann et al. (2018) in patients with poorly controlled diabetes reported a significant reduction in both hypoglycaemia (from 3.9% to 2.6%) and hyperglycaemia (from 42% to 32%) and increased time-in-range (from 54% to 65%) with the hybrid system compared with standard insulin therapy. This study highlights how hybrid systems may also benefit patients with sub-optimally controlled diabetes. 

Despite these advantages, there are several limitations to consider for hybrid systems. Primarily, their partial automation means users must still initiate boluses to account for variations such as meals and exercise, which increases user burden. As postprandial glycaemia is primarily driven by rapid rises in glucose which cannot be countered by basal insulin alone, delay or failure to administer boluses can result in significant glycaemic deviations. As such, the extent to which these systems replicate endogenous pancreatic physiology is limited. 

Nevertheless, hybrid closed-loop systems represent a transformative step in diabetes management, and improvements in both clinical and psychosocial measures have been demonstrated across a range of populations. Despite falling short of fully replicating healthy pancreatic physiology, these systems provide a bridge between traditional insulin pump therapy and a fully autonomous and physiological artificial pancreas.

Fully Closed Loop Systems 

Fully closed-loop systems (CLSs), often described as the ‘holy grail’ of artificial pancreas design, aim to incorporate automated prandial insulin boluses into the algorithm to complement basal insulin delivery without any user intervention. By automating meal-time dosing and adapting dynamically to daily life variables such as activity levels, sleep and stress, the goal of these systems is to fully replicate the regulatory capacity of pancreatic b-cells.

Although commercially available FCLs remain limited, multiple clinical trials have demonstrated promising results. These systems integrate CGM with sophisticated algorithms to detect mealtime glucose excursions and deliver appropriate prandial insulin doses, either via prediction or detection algorithms [28]. Early trials focused primarily on overnight glycaemic control, with results consistently demonstrating improved glycaemic control [29-32] For instance, Hovorka et al. (2014) demonstrated in a crossover study involving 16 adolescents that an FCL system reduced hyperglycaemia significantly (from 16.2% to 9.5%) and increased time-in-range from 69% to 85% overnight, compared with conventional insulin pump therapy [29]. 

Likewise, Haidar et al. (2015) reported similar results in children with increased time in range (from 54% to 77%) and reduced time in hyperglycaemia (from 38% to 20%) overnight [30]. However, in both these studies, time in hypoglycaemia was not significantly different between the study groups, highlighting a shortfall in early FCL system algorithms. 

Clinical trials involving daytime control also demonstrate improved glycaemic outcomes [31]; however, the results are more variable. For instance, Reddy et al. (2016) observed a significant reduction in overall hypoglycaemia (from 17.9% with insulin pump therapy to 3% with the FCLS) but reported an increase in 24-hour and nighttime hyperglycaemia with the FCLS. As such, this study highlighted that some FCLS algorithms may prioritise avoidance of hypoglycaemia, especially overnight, by potentially under-delivering insulin during periods of hyperglycaemia. This means that whilst FCL systems might reduce the risk of dangerous lows, they still face limitations in postprandial and daytime glycaemic control - partly due to delays in subcutaneously delivered insulin.

This core issue, the pharmacokinetic lag of even rapid-acting insulin analogues, remains the main limiting factor of most fully automated systems. Compared with endogenous insulin secretion, insulin analogues have a delayed onset and prolonged duration of action [33,34]. This gap is exacerbated by the lag between physiological lag between glucose concentrations in the interstitial and vascular spaces [35]. As a result, the FCL systems often fail to replicate the rapid insulin secretion profiles required for precise control, especially postprandially and during exercise. 

Some systems attempt to compensate with ‘aggressive insulin modes’, which deliver larger boluses when glucose rises rapidly. These systems aim to compensate for delays in infusion and action to mimic the first stage of physiological insulin secretion [36]. However, this strategy increases hypoglycaemia risk and, as such, requires a safety feedback controller to replicate β-cell suppression [37]. 

Alternative approaches have also recently emerged to manage postprandial challenges. One possibility is the incorporation of a meal anticipation algorithm which estimates carbohydrate intake based on past meal timing or glucose trends, triggering preemptive insulin dosing. Small-scale clinical trials have demonstrated potential for this system; for instance, a randomised crossover study by Palisaitis et al. (2021) reported improved glycaemic control with reduced time spent in hyperglycaemia (58.0% compared with 79.6%) with the algorithm. However, the mean detection time was 40 minutes, indicating a delay in response [38]. Additionally, these methods remain vulnerable to errors from sensor inaccuracies or unannounced food intake.

Recent advancements have partially overcome these limitations via the incorporation of mealtime announcements. In these systems, the user gives a pre-meal signal, and the algorithm handles the rest [28]. Most notably, the Beta Bionics iLet Bionic Pancreas (insulin-only configuration) was approved by the FDA in 2023 and has demonstrated improved glycaemic outcomes, including improved time in range and reduced glycated haemoglobin levels [39]. In this trial, users only needed to indicate meal size (small, medium, or large), significantly reducing the burden of diabetes management whilst improving outcomes. However, notably this trial also reported an increased rate of hyperglycaemic adverse events with the bionic pancreas compared with the standard care group (214 vs. 2). This was related to infusion-set failure. Furthermore, two participants required adjunctive insulin glargine with the bionic pancreas due to prolonged hyperglycaemia despite exceeding maximum insulin allowances by the algorithm. As such, this reiterates the core limitation of FCL systems being the delayed pharmacokinetics of current insulin analogues. 

Other nuanced approaches include bioinspired models, such as those which simulate biphasic insulin secretion or use physiological feedback mechanisms to prevent insulin stacking. Pilot trials have highlighted the potential to reduce nighttime hypoglycaemia, but often at the cost of increased postprandial hyperglycaemia [40]. Additionally, fuzzy logic has been utilised for FCL insulin delivery to replicate caregiver decision-making. By encoding rules based on user traits (e.g., insulin sensitivity, activity levels, regular meals), fuzzy logic allows context-specific algorithm modulation. Whilst feasibility has been demonstrated in a controlled setting [20], widespread clinical application is still pending. 

In summary, fully closed-loop systems represent a critical next step in artificial pancreas development. Recent advancements, such as the iLet Bionic Pancreas, represent major breakthroughs; however, limitations regarding postprandial hyperglycaemia persist due to delayed insulin absorption. Future strategies focusing on ultrafast insulin analogues, alternative delivery methods, or adjunct therapies could further enhance FCL system performance. 

Dual Hormone Systems

Dual-hormone APSs represent the next evolutionary step in mimicking true endocrine pancreatic physiology. By combining automated delivery of glucagon alongside insulin, these systems aim to not only correct hyperglycaemia but also prevent or rapidly correct hypoglycaemia, something that insulin-only systems inherently struggle with due to the delayed pharmacokinetic profile of insulin analogues [41].

In healthy individuals, glucagon is secreted by pancreatic α-cells in response to falling glucose levels and acts to promote hepatic glucose production. In T1DM this glucagon response is often impaired or absent, significantly increasing the risk of hypoglycaemia, particularly in children, individuals with long-standing diabetes where glucagon secretory capacity is impaired, or those with hypoglycaemia unawareness [42]. 

Clinical trials of dual-hormone systems have consistently demonstrated that adding glucagon reduces the incidence of hypoglycaemia compared with insulin-only systems, particularly in high-risk or high-activity scenarios such as exercise and overnight periods [30,43-46]. For instance, Haidar et al. (2015) observed that the dual-hormone system reduced overnight hypoglycaemia from 3.1% to 0% in children, with a corresponding reduction in mean glucose levels, indicating improved safety without sacrificing control overnight [30]. 

Likewise, Taleb et al. (2016) evaluated the performance of a dual-hormone artificial pancreas during a controlled exercise challenge and found that it eliminated hypoglycaemia, compared to an 11% time-in-hypoglycaemia observed with standard insulin therapy [44]. Building on this, Castle et al. (2018) conducted a four-day outpatient trial that included multiple exercise sessions. Their findings confirmed that the dual-hormone system consistently reduced hypoglycaemic episodes both during physical activity and over the entire study period [46]. These results highlight the value of glucagon in managing exercise-induced glycaemic variability, an area where insulin-only systems struggle due to the delayed onset of subcutaneous insulin action. Insulin suspension alone is often too slow to counteract rapid glucose declines, and relying on carbohydrate intake can lead to overshooting and rebound hyperglycaemia. Importantly, neither study reported an increase in overall hyperglycaemia or mean glucose levels, suggesting that the addition of glucagon provides a meaningful safety benefit without compromising glycaemic control [47].

Mechanistically, the rapid onset of glucagon makes it well-suited for short-term glucose support. Administered in microdoses, it can raise glucose levels within minutes and is particularly beneficial when insulin stacking or delayed carbohydrate absorption threatens to induce hypoglycaemia [48]. Furthermore, the inclusion of glucagon allows algorithms to adopt more aggressive insulin dosing strategies without the same hypoglycaemic risk, thereby improving postprandial control as well. However, these benefits are accompanied by several limitations, notably the increased technical complexity, glucagon instability and long-term safety. Current dual-hormone systems require two pumps and infusion sets, which increases the complexity and burden of wear, as well as increases the risk of mechanical failure. Moreover, commercially available glucagon is prone to degradation in aqueous solution, necessitating daily reconstitution or the use of specialised stabilising formulations [47]. As such, this limits real-world usability. Whilst more stable analogues (e.g., dasiglucagon) are under investigation, integration into closed-loop systems has not been well established [49]. Additionally, clinical trials of dual-hormone systems have been short in duration, and as such, the long-term effects of chronic low-dose glucagon administration, particularly regarding hepatic glycogen depletion, nausea or desensitisation, remain incompletely understood [50].

Despite their current limitations, dual-hormone systems represent a significant advance towards the development of a truly biomimetic artificial pancreas. By replicating the coordinated actions of both insulin and glucagon, these systems more closely resemble the hormonal feedback loop which maintains stable blood glucose levels. Looking ahead, innovations in glucagon formulation, such as ultra-stable analogues, and the development of integrated dual-chamber pump platforms may enhance both the practicality and scalability of these systems, making them more accessible to a broader patient population.

The 'Designer' Artificial Pancreas

The concept of a ‘Designer’ Artificial Pancreas envisions a system which is not only physiologically intelligent but also user-friendly, personalised and can be seamlessly integrated into daily life. Such a system would require minimal user input, dynamically adapt to the individual's changing glycaemic patterns, and cope with real-world contexts such as meals, exercise, sleep and stress. 

Current APS technologies, whilst transformative, remain imperfect, and several limitations persist. A significant barrier to full automation includes the delayed pharmacokinetics of subcutaneous insulin analogues, which greatly limits the system’s ability to handle rapid postprandial glucose excursions. Even the fastest available insulin formulations cannot match the immediate first-phase insulin secretion of a healthy pancreas. As a result, most commercial and investigational systems still require user-initiated meal boluses or basic meal announcements to help counter this limitation.

Several strategies have been proposed to overcome this challenge, including improved insulin analogues with faster onset of action. For instance, faster-acting aspart and ultra-rapid lispro demonstrate faster absorption and action time and have been shown to improve postprandial control and reduce insulin stacking by earlier inhibition of hepatic glycogenolysis [51]. Additionally, adjunct approaches including local warming devices can further accelerate absorption by increasing local blood flow; however, user burden remains a concern [52]. 

Alternative strategies which have been proposed include alternative delivery routes such as inhaled or oral insulins which effectively bypass the subcutaneous tissue entirely and thus achieve a more rapid systemic response. However, these are yet to be successfully integrated into a closed-loop system due to variability in absorption, delivery logistics and limited dose flexibility [51]. 

Adjunctive therapies such as pramlintide, an amylin analogue, present another strategy. By slowing gastric emptying and suppressing postprandial glucose release, pramlintide can attenuate the rate and magnitude of glucose appearance following meals, potentially improving glycaemic stability when used alongside insulin. However, integration into a feedback-controlled system remains challenging. By delaying a rise in glucose levels, it might act to reduce insulin delivery if the algorithm misinterprets the delayed glucose excursion, potentially undermining the intended benefit [53]. 

Finally, as we discussed earlier, some investigational systems are developing AI-driven prediction models which identify patterns in meal timing and glucose kinetics to deliver insulin preemptively. Whilst early trials showed improvement in postprandial glucose control in adolescents prone to missed insulin boluses, these systems remain vulnerable to unannounced meals and sensor error, often requiring refinement to prevent mistimed dosing or hypoglycaemia [38]. 

Beyond pharmacology, algorithm personalisation may help more accurately replicate the physiological insulin delivery profile of the β-cell. For instance, the incorporation of multiple interacting signals, such as cortisol, sex hormones, heart rate variability, or sleep dynamics, into algorithms could more accurately reflect the fluctuating insulin requirements. By quantifying the effects of these inputs on blood glucose regulation, current algorithms could be modified to enable dynamic personalised dosing [53].

Importantly, a ‘designer’ APS must also address the psychosocial factors associated with living with diabetes. While many users welcome automatic and reduced decision-making burdens, others prefer to maintain control or customise aspects of their therapy. Overall, studies have consistently reported increased quality of life, reduced anxiety and greater confidence with APS use [4]. However, issues are also highlighted, including alarm intrusiveness, device size and trust in automation, which may be reduced with longer periods of use [19].

A truly designer system, therefore, may not be a one-size-fits-all but rather an adaptable and dynamic platform which supports multiple modes and degrees of interaction. Customisable algorithms, optional user overrides, and flexible control settings could allow users varying degrees of autonomy so that they can tailor their interaction based on lifestyle, confidence and need. By achieving this, the artificial pancreas would evolve beyond a glycaemic control device to become a responsive and collaborative partner in personalised diabetes management.

Future Implications

While the ultimate solution for Type 1 diabetes lies in biological cures, such as β-cell replacement or immunotherapy, the artificial pancreas remains the most promising near-term tool to transform daily diabetes management. Over the past decade, rapid advances in sensor technology, insulin delivery, and algorithm design have moved APSs from research prototypes to commercial reality. Yet, despite this progress, no current system fully replicates the responsiveness, precision, or adaptability of the native pancreas.

Hybrid systems have demonstrated clear clinical benefits and strong user acceptance but rely heavily on manual input for meals. Fully closed-loop systems offer greater automation, particularly overnight, but continue to struggle with postprandial control due to insulin pharmacokinetics. Dual-hormone systems show the greatest physiological fidelity by incorporating glucagon to counteract hypoglycaemia, though they remain limited by practical challenges such as pump complexity and glucagon stability.

The next generation of APSs, the so-called “designer” systems, will need to marry robust automation with flexibility, personalisation, and minimal user burden. Advances in ultra-rapid insulin analogues, stable glucagon formulations, multi-hormonal delivery systems, and machine learning algorithms are all converging to bring this vision closer to reality. Equally important will be attention to human factors, for instance, intuitive interfaces, customisable modes of engagement, and adaptive control options to allow personalisation and varying degrees of autonomy.

Crucially, innovation is not limited to industry. The DIY diabetes technology community has already demonstrated the feasibility of user-led closed-loop systems, such as OpenAPS and Loop, showing what’s possible when patients take design into their own hands. These efforts reflect a growing recognition that the most effective artificial pancreas is not merely one that automates insulin but one that aligns with the lived experience of diabetes.

## Conclusions

The ideal artificial pancreas system would operate without the need for manual input or carbohydrate counting, fully replicating endogenous insulin and glucose profiles. While no current system fully achieves this vision, it remains a central goal driving ongoing research. 

Dual-hormone systems come closest to replicating physiological pancreatic function; however, their added complexity may limit broad applicability and make them best suited for subpopulations at high risk of hypoglycaemia. Crucially, questions around cost-effectiveness, infrastructure demands, and training requirements persist, and will need to be addressed through large-scale clinical trials that assess performance and feasibility across diverse patient populations. 
